# Effects of early corticosteroid use in patients with severe coronavirus disease 2019

**DOI:** 10.1186/s12879-021-06221-5

**Published:** 2021-05-31

**Authors:** Jong Hoon Hyun, Moo Hyun Kim, Yujin Sohn, Yunsuk Cho, Yae Jee Baek, Jung Ho Kim, Jin Young Ahn, Jun Yong Choi, Joon Sup Yeom, Mi Young Ahn, Eun Jin Kim, Ji-Hyeon Baek, Young Keun Kim, Heun Choi, Su Jin Jeong

**Affiliations:** 1 Department of Internal Medicine, Severance Hospital, Yonsei University College of Medicine, 50-1 Yonsei-ro Seodaemun-gu, Seoul, 03722 South Korea; 2 Department of Internal Medicine, Seoul Medical Center, Seoul, South Korea; 3 Department of Infectious Diseases, Ajou University School of Medicine, Suwon, South Korea; 4 Department of Internal Medicine, Inha University School of Medicine, Incheon, South Korea; 5 Department of Internal Medicine, Yonsei University Wonju College of Medicine, Wonju, South Korea; 6 Department of Infectious Disease, National Health Insurance Service Ilsan Hospital, Goyang, South Korea

**Keywords:** Coronavirus disease 2019, COVID-19, SARS-CoV-2, Pneumonia, Corticosteroid

## Abstract

**Background:**

Coronavirus disease 2019 (COVID-19) is associated with acute respiratory distress syndrome, and corticosteroids have been considered as possible therapeutic agents for this disease. However, there is limited literature on the appropriate timing of corticosteroid administration to obtain the best possible patient outcomes.

**Methods:**

This was a retrospective cohort study including patients with severe COVID-19 who received corticosteroid treatment from March 2 to June 30, 2020 in seven tertiary hospitals in South Korea. We analyzed the patient demographics, characteristics, and clinical outcomes according to the timing of steroid use. Twenty-two patients with severe COVID-19 were enrolled, and they were all treated with corticosteroids.

**Results:**

Of the 22 patients who received corticosteroids, 12 patients (55%) were treated within 10 days from diagnosis. There was no significant difference in the baseline characteristics. The initial PaO_2_/FiO_2_ ratio was 168.75. The overall case fatality rate was 25%. The mean time from diagnosis to steroid use was 4.08 days and the treatment duration was 14 days in the early use group, while those in the late use group were 12.80 days and 18.50 days, respectively. The PaO_2_/FiO_2_ ratio, C-reactive protein level, and cycle threshold value improved over time in both groups. In the early use group, the time from onset of symptoms to discharge (32.4 days vs. 60.0 days, *P* = 0.030), time from diagnosis to discharge (27.8 days vs. 57.4 days, *P* = 0.024), and hospital stay (26.0 days vs. 53.9 days, *P* = 0.033) were shortened.

**Conclusions:**

Among patients with severe COVID-19, early use of corticosteroids showed favorable clinical outcomes which were related to a reduction in the length of hospital stay.

## Background

Coronavirus disease 2019 (COVID-19), a disease caused by severe acute respiratory syndrome coronavirus 2 (SARS-CoV-2), was first reported in China in late 2019 [1]. It has affected almost 25 million people worldwide, and resulted in the deaths of more than 850,000 as of August 31, 2020 [2]. The majority of infected patients are asymptomatic and show only mild symptoms; however, the remainder of patients experience a severe form of the disease. Elderly patients and patients with underlying diseases, such as diabetes mellitus, hypertension, and immunosuppressive disorders, have considerable morbidity and mortality when co-infected with COVID-19 [3, 4]. The overall fatality rate is approximately 0.4–2.0%, but may be up to 50% in patients with life-threatening illnesses [5], a finding which suggests that it is necessary to use different treatment approaches in these patients.

Severe COVID-19 is accompanied by inflammatory organ injury and causes acute respiratory distress syndrome, shock, or cardiac failure due to elevated levels of inflammatory cytokines and biomarkers. These include C-reactive protein (CRP), erythrocyte sedimentation rate (ESR), ferritin, and D-dimer [6, 7]. Although the main pathophysiology of COVID-19 is not completely understood, excessive inflammation is closely related to the development of pneumonia and rapid progression of the disease [6, 8]. Anti-inflammatory treatments, such as corticosteroids, are therefore, therapeutic options for severe COVID-19 [9].

Several potential therapeutics have been proposed in the early phase of the COVID-19 pandemic, such as chloroquine, hydroxychloroquine, lopinavir/ritonavir, azithromycin, intravenous immune globulin, and convalescent plasma transfusion; but corticosteroids and remdesivir have been known to improve clinical outcome so far [10, 11].. Corticosteroid, especially methylprednisolone, have been widely used in patients with severe COVID-19 cases in South Korea before the WHO and CDC made recommendations against using corticosteroid. Although some studies have shown that the usefulness of corticosteroid is limited [12–14], it has still become a potentially good therapeutic option as other studies, including the RECOVERY trial [11], have demonstrated beneficial effects of corticosteroid use [15–18]. The RECOVERY trial is a large-scale, prospective, well-designed study, which demonstrated that the use of dexamethasone reduces mortality in patients with severe COVID-19 [11]. Other types of corticosteroids, such as hydrocortisone [19], have also been demonstrated to improve clinical outcomes [20]. The use of corticosteroids could theoretically cause several side effects [21], but did not inhibit secondary bacterial infection or viral clearance [22, 23]. Strong evidence for dexamethasone use has been suggested from the RECOVERY trial. Because this trial has strong evidence for corticosteroid use, relatively fewer other studies were conducted. As a result, there is insufficient data to determine which types of corticosteroid (dexamethasone, hydrocortisone, methylprednisolone) are more beneficial [20], and the appropriate duration [24] and timing to start corticosteroid use still remain controversial.

This study aimed to analyze the results in groups with early and late use of corticosteroids to determine the right timing of corticosteroid use in patients with severe COVID-19.

## Methods

### Study design and population

This was a retrospective cohort study of patients with severe cases of COVID-19 who received corticosteroid treatment between March 2 and June 30, 2020 in seven tertiary hospitals in South Korea. The current study included patients aged ≥18 years with laboratory-confirmed SARS-CoV-2 infection who were admitted to the ICU. Patients with insufficient clinical data due to hospital transfers were excluded.

Electronic medical records were reviewed for baseline demographics, comorbidities, clinical characteristics, clinical status, laboratory findings, treatment, clinical course, and outcomes. Data were compared before steroid use, as well as 3, 7, and 14 days later.

This study was conducted in accordance with the guidelines of the Declaration of Helsinki and approved by the Institutional Review Boards of Severance Hospital (Seoul, South Korea). The requirement for informed consent was waived because of the retrospective nature of the study. All data was anonymized to keep the confidentiality of the patient’s personal information during the entire research process including patient data collection, analysis and writing.

### Definition

SARS-CoV-2 RNA was assessed by real-time reverse transcription polymerase chain reaction (RT-PCR) of nasopharyngeal swabs or sputum according to the World Health Organization interim guidance. RT-PCR assays for the *E*, *RdRp*, and *N* genes were performed using the Allplex™ 2019-nCoV Assay (Seegene Inc., Seoul, South Korea). Positive RT-PCR results were defined as a cycle threshold (Ct) value ≤40. The severity was assessed by PaO_2_/FiO_2_ ratio and categorized into seven scores based on oxygen supplementation: no limit of activity, limit of activity but no O_2_, O_2_ with nasal prong, O_2_ with facial mask, high flow nasal cannula, noninvasive ventilation, and invasive ventilation.

The patients were divided into two groups by the time to corticosteroid use. Patients who were treated with corticosteroids within 10 days and after 10 days of confirmation were categorized as the early use group and the late use group, respectively.

CRP concentration was measured in serum using a nephelometric method (Beckman Coulter, Fullerton, CA, USA).

### Statistical analysis

Continuous variables were examined for normality by Kolmogorov-Smirnov test. Continuous variables with normal distribution were shown as the mean ± standard deviation (SD). Group comparisons were performed using independent two-sample t-test. Continuous non-normal distribution variables were shown as the median interquartile range (3rd interquartile range-1st interquartile range), and Mann-Whitney U tests were used to compare the differences between groups. Categorical variables were shown as numbers (percentages). Chi-squared tests and Fisher’s exact test were used to compare categorical data in different groups. Laboratory findings were analyzed based on a linear mixed model using groups (early or late) and time (after corticosteroid administration) and an interaction between groups and time. Spearman’s correlation coefficient was calculated to analyze correlation of variables. Statistical significance was set at *P* < 0.05. All statistical analyses were performed using the Statistical Package for the Social Sciences version 25.0 (IBM Corporation, Armonk, NY, USA).

## Results

During the study period, 22 patients who met the inclusion criteria were categorized into the early use group (*n* = 12) or the late use group (*n* = 10). The baseline demographics and characteristics of each group were similar (Table 1). The mean age of the early use group was 65.6 years, and 50% were male. Among them, seven patients (58.3%) had a history of hypertension. The most common symptoms were fever (75%), cough (50%), sputum (41.7%), dyspnea (41.7%), and fatigue (41.7%). Patients were assessed based on an ordinal scale; four patients (33.3%) had a score of 2 (limit of activity but no O_2_); five (41.7%) had a score of 3 (O_2_ with nasal prong), and three (25%) had a score of 5 (high flow nasal cannula). Baseline laboratory tests were similar between the two groups. The initial PaO_2_/FiO_2_ ratio in the early use group was 124.89. The inflammatory markers were elevated; the mean levels of ferritin, ESR, and CRP were 804.22 ± 601.11 ng/mL, 60.00 ± 35.78, and 10.33 ± 8.95, respectively.

**Table 1:** Comparisons of baseline demographics and characteristics in patients with severe COVID-19

Characteristics	Early use group (***n*** = 12)	Late use group (***n*** = 10)
**Age (years) mean ± SD**	65.6 ± 5.6	74.6 ± 4.6
**Sex, No. (%)**		
Male	6/12 (50%)	6/10 (60%)
**Coexisting disease, No. (%)**		
Hypertension	7/12 (58.3%)	4/10 (30%)
Diabetes	3/12 (25%)	3/10 (30%)
Malignant neoplasm	2/12 (16.7%)	1/10 (10%)
**Symptoms**		
Fever	9/12 (75%)	7/10 (70%)
Cough	6/12 (50%)	3/10 (30%)
Sputum	5/12 (41.7%)	4/10 (40%)
Dyspnea	5/12 (41.7%)	4/10 (40%)
Myalgia	5/12 (41.7%)	2/10 (20%)
Fatigue	5/12 (41.7%)	1/10 (10%)
Poor oral intake	4/12 (33.3%)	1/10 (10%)
**Initial score on ordinal scale**		
No limit of activity	0	0
Limit of activity but no O_2_	4/12 (33.3%)	4/10 (40%)
O_2_ with nasal prong	5/12 (41.7%)	2/10 (20%)
O_2_ with facial mask	0	0
High flow nasal cannula	3/12 (25%)	3/10 (30%)
Non-invasive ventilation	0	0
Invasive ventilation	0	0
Baseline score missing	0	1/10 (10%)
**Initial PaO2/FiO**_**2**_ **ratio**	124.89 ± 42.60	133.35 ± 49.40

COVID-19, coronavirus disease 2019; SD, standard deviation

Data are expressed as mean ± SD or number (%)

The therapeutic options and timing differences of patients are shown in Table 2. Most patients used methylprednisolone, and only one patient used hydrocortisone. Corticosteroids were initiated within a median of 9.75 ± 3.64 days of the onset of symptoms, 3.00 (2.00–7.00) days of the confirmation, and 3.33 ± 3.45 days after hospital admission. The initial dose of corticosteroid administered was 0.81 (0.50–1.00) mg kg^− 1^ day^− 1^ methylprednisolone, and patients were treated for 14.00 ± 5.00 days. The total dose administered was 521.6 ± 246.38 mg methylprednisolone.

**Table 2:** Factors associated with therapeutic option in patients with severe COVID-19

	Early use group (***n*** = 12)	Late use group (***n*** = 10)	***p***-value
**Combination therapy**			
Steroid	7/12 (58.3%)	8/10 (80.0%)	0.162
Steroid + Convalescent plasma	5/12 (41.7%)	1/10 (10.0%)	
Steroid + Remdesivir	0	1/10 (10.0%)	
**Steroid-related factor**			
Time from symptom to steroid use	9.75 ± 3.64	15.70 ± 6.00	0.010
Time from diagnosis to steroid use	3.00 (2.00–7.00)	12.5 (11.00–14.25)	< 0.001
Time from hospitalization to steroid use	3.33 ± 3.45	10.00 ± 4.83	0.001
Initial dose^a^, mg/kg/day	0.81 (0.50–1.00)	1.00 (0.76–.1.00)	0.456
Duration, days	14.00 ± 5.00	18.50 ± 14.76	0.380
Total dose^a^, mg	521.6 ± 246.38	667.50 ± 606.76	0.490
**Score on ordinal scale at steroid start**			0.035
O_2_ with nasal prong	1/8 (12.5%)	0	
High flow nasal cannula	7/8 (87.5%)	2/5 (40%)	
Invasive ventilation	0	3/5 (60%)	

COVID-19, coronavirus disease 2019

Continuous variables were examined for normality by Kolmogorov-Smirnov test. Continuous variables with normal distribution are shown mean ± standard deviation (SD). Group comparisons were performed using independent two-sample t-test. Continuous non-normal distribution variables are shown as the median interquartile range, and Mann-Whitney U tests were used to compare the differences between groups. Categorical variables are shown as numbers (percentages). Chi-squared tests and Fisher’s exact test were used to compare categorical data in different groups

^a^Dose of corticosteroid was calculated based on methylprednisolone

**Fig. 1:**
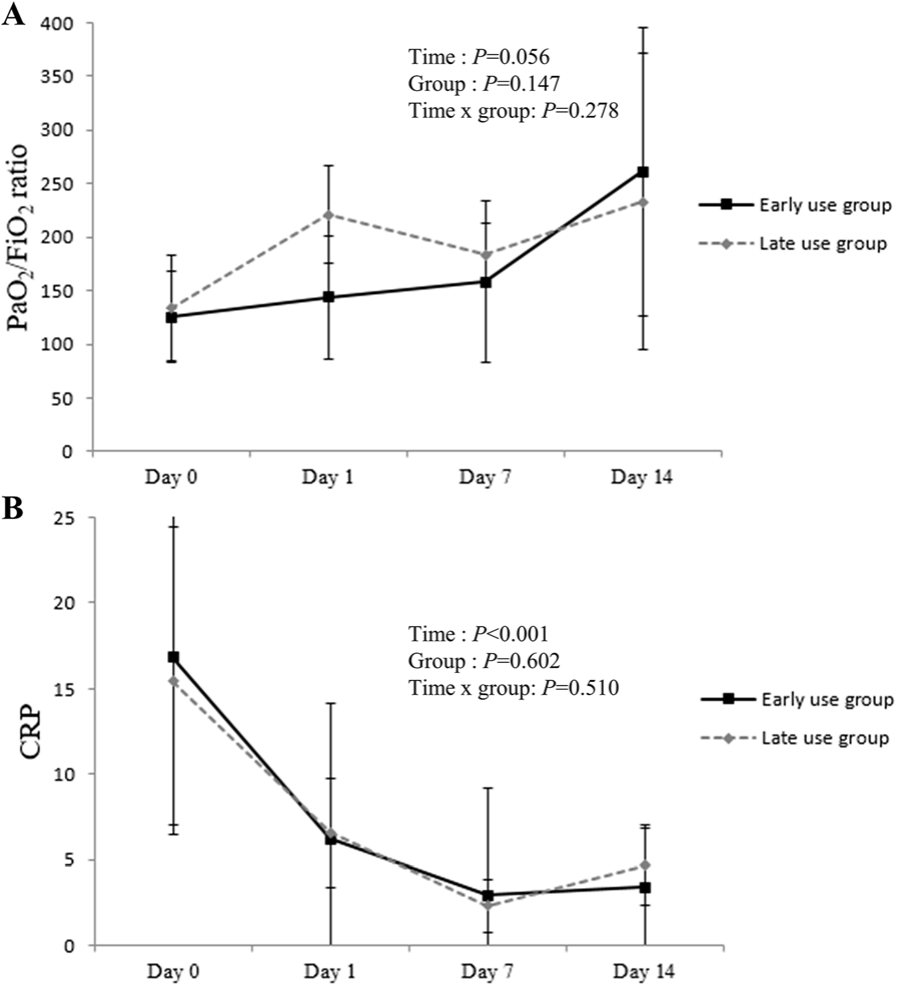
Comparison of clinical response after corticosteroid use. **A** Changes in mean PaFiO2 ratio **B** Changes in mean CRP. Means were calculated and compared between groups at each time point using a linear mixed model. Error bars represent standard error. CRP, C-reactive protein

Table 3 shows the comparison of laboratory findings before and after corticosteroid use. Significant changes in lymphocyte count, lactate dehydrogenase, and inflammatory markers (ferritin, CRP, and procalcitonin) were observed after corticosteroid administration in both groups. The PaO_2_/FiO_2_ ratio and Ct value improved in both groups. However, when comparing changes between the two groups, there was no significant difference in improvement (Fig. 1).

**Table 3:** Change in laboratory findings after corticosteroid use

	Early use group (n = 12)				Late use group (n = 10)			
	Day 0	Day 3	Day 7	Day 14	Day 0	Day 3	Day 7	Day 14
WBC count, cells/μL	7496.67 ± 4065.33	9083.33 ± 3157.70	11,343.64 ± 5408.91	9762.00 ± 3989.17	8330.00 ± 2953.13	9738.00 ± 2988.52	12,063.00 ± 3200.34	10,045.56 ± 4728.95
Neutrophil count, cells/μL	706.75 ± 3451.137	7999.33 ± 2943.50	9954.64 ± 5162.74	7918.40 ± 4030.34	6918.10 ± 2844.49	6878.24 ± 2988.34	10,142.90 ± 3488.86	8444.31 ± 4657.33
Lymphocyte, cells/μL	756.77 ± 280.28	653.5 ± 247.79	830.20 ± 525.49	901.80 ± 446.39	774.86 ± 31.49	778.2 ± 535.66	906.40 ± 640.20	803.22 ± 451.07
LDH, IU/L	444.30 ± 113.95	366.75 ± 99.70	363.63 ± 95.43	339.43 ± 179.34	394.63 ± 126.97	463.60 ± 201.04	402.63 ± 157.78	367.50 ± 200.63
Ferritin, ng/mL	1423.8 ± 1047.96	966.08 ± 765.58	841.28 ± 465.38	1236.91 ± 975.47	1045.37 ± 895.92	1252.22 ± 1166.20	698.31 ± 167.39	813.22 ± 420.80
CRP, mg/L	16.83 ± 9.76	6.25 ± 7.93	2.95 ± 6.28	3.40 ± 3.44	15.42 ± 8.97	6.61 ± 3.18	2.33 ± 1.58	4.70 ± 2.34
Procalcitonin, ng/mL	0.33 ± 0.28	0.49 ± 0.47	0.17 ± 0.13	0.15 ± 0.25	0.54 ± 0.95	0.23 ± 0.11	0.11 ± 0.03	0.11 ± 0.06
PaO_2_/FiO_2_ ratio	124.89 ± 42.60	143.42 ± 57.61	157.70 ± 75.48	261.09 ± 134.38	133.35 ± 49.40	220.97 ± 45.61	183.40 ± 29.92	232.77 ± 138.59
Ct value	24.77 ± 8.42	27.31 ± 5.91	29.3 ± 8.85	32.73 ± 5.71	31.5 ± 3.07	24.53 ± 1.31	28.30 ± 7.14	35.40 ± 2.46

*WBC* white blood cell, *LDH* lactate dehydrogenase, *CRP* C-reactive protein, *Ct value* cycle threshold

Data are expressed as mean ± SD

**Fig. 2:**
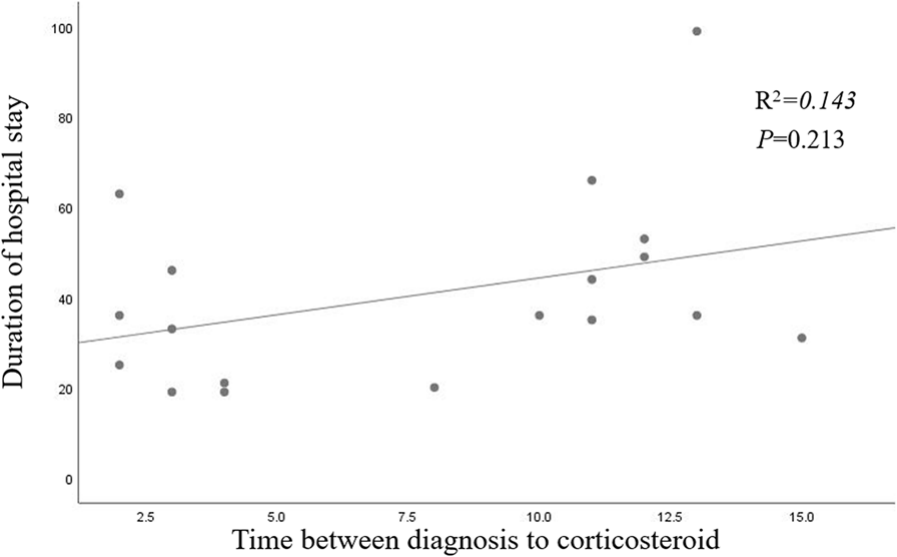
Correlation between time of steroid use and duration of hospital stay in the early use group

Table 4 shows the comparison of clinical outcomes between the two groups. The duration of hospital stay was shorter in the early use group than in the late use group (26.0 ± 11.4 vs. 53.9 ± 23.0 days, *P* = 0.033). The time from corticosteroid use to discharge was 25.6 days in the early use group and 46.3 days in the late use group, but the difference was not significant (*P* = 0.096). We could not demonstrate a correlation between the time between diagnosis and corticosteroid use, and the duration of hospital stay (R^2^ = 0.143, *P* = 0.213) (Fig. 2). There was no significant difference in the overall mortality. None of the patients in either group suffered secondary bacterial infection or hyperglycemia.

**Table 4:** Clinical outcomes in the early use and late use groups

	Early use group (***n*** = 12)	Late use group (***n*** = 10)	***p***-value
Time from diagnosis to discharge	27.8 ± 12.2	57.4 ± 22.5	0.024
Time from symptom to PCR-negative	25.0 (22.0–38.0)	37.5 ± (28.0–50.0)	0.190
Time from symptom to discharge	32.4 ± 13.1	60.0 ± 21.5	0.030
Time from corticosteroid use to discharge	25.6 ± 12.4	46.3 ± 22.7	0.096
Length of hospital stay	26.0 ± 11.4	53.9 ± 11.4	0.033
Overall mortality, No./Total (%)	1/12 (8.3%)	3/10 (30%)	0.293

*PCR* polymerase chain reaction

Continuous variables with normal distribution are shown as mean ± SD. Group comparisons were performed using independent sample t-test. Continuous non-normal distribution variables are shown as median interquartile range. and Mann-Whitney tests were used to compare the difference between groups

## Discussion

The COVID-19 pandemic has been worsening and spreading worldwide. Although many types of treatment, including chloroquine, hydroxychloroquine, lopinavir/ritonavir, azithromycin, intravenous immune globulin, and convalescent plasma transfusion, have been used in the early phase of COVID-19 pandemic, they have been proven to be ineffective and are no longer used. An antiviral agent like Remdesivir [11], as well as immunomodulatory agents such as Tocilizumab (IL-6 inhibitor) [25] and Baaricitinib (JAK inhibitor) [26], and monoclonal antibodies like Bamlanivimab [27], and Casirivimab/imdevimab [28] can be used to treat patients with COVID-19. However, these types of medicine have been found to be less useful in critically ill patients with COVID-19, so additional treatment options are still needed for patients with severe COVID-19.

Corticosteroids have long been considered to be effective in patients with acute respiratory distress syndrome, septic shock, and possibly viral pneumonia [29]; but this is still controversial. An interim guidance document released on May 27, 2020 by the World Health Organization on clinical management of COVID-19 recommended against the routine use of systemic corticosteroids for the treatment of viral pneumonia. The Infectious Disease Society of America recommends the use of corticosteroids in the context of clinical trials for patients with acute respiratory distress syndrome [30]. However, adherence to this recommendation remains low, with many studies suggesting opposite results. Studies favoring the use of corticosteroids began with the RECOVERY trial, which demonstrated that intravenous dexamethasone (6 mg daily for 10 days) reduced 28-day mortality in hospitalized patients with COVID-19 requiring oxygen therapy or mechanical ventilation [11]. This landmark trial has been supported by another trial [19, 31, 32] and meta-analysis [15, 17, 18, 33]. These studies provide evidence on the effectiveness of corticosteroid, suggest a safe treatment option for COVID-19, and have changed our clinical practice regarding patients with severe COVID-19.

The pathogenesis of severe COVID-19 still remains unclear, but it is believed to have two overlapping pathologic subsets, similar to other types of viral pneumonia. In the early stage of infection, as SARS-CoV-2 replicates, mild clinical manifestations such as fever, malaise, and cough are observed. In most of these cases, patients recover without therapeutic support; but in other cases, they progress to severe disease. This is a result of host systemic inflammation rather than direct viral-induced tissue damage. Since sepsis and other critical illnesses occur for approximately 10 days [3], the maladaptive host response to the viral infection starts during this period. For this reason, anti-inflammatory therapy, such as corticosteroids, is not recommended for early use. Many studies have shown that corticosteroids can prolong viral shedding and cause secondary infections [34–36]. In our study, patients were divided into two groups based on the duration from diagnosis to corticosteroid use on a 10-day basis. Both groups showed increasing Ct values after corticosteroid use. Within each group, the increase was significant, but there was no difference between the two groups. Therefore, corticosteroid can be free from the stigma of inhibition of viral clearance for using corticosteroids in patients with severe COVID-1. Our findings were consistent with the results of a recent study [22].

As corticosteroids generally suppress the immune system response, there is a concern that they can accelerate secondary infections and cause many complications such as diabetes, psychosis, and avascular necrosis [36]. In our study, we assessed the patients’ initial severity and response to corticosteroid using procalcitonin, CRP, and LDH, as shown in Table 3, which were relatively well-studied regarding the predictability of severity [7]. Patients with lymphopenia recovered, and inflammatory parameters such as ferritin, CRP, and procalcitonin also improved after corticosteroid use in both groups, although there was no difference between the two groups. Corticosteroid-related complications were not noted.

The mean length of hospital stay for COVID-19 patients varies from 4 to 11 days, and those with severe and life-threatening cases of COVID-19 remain hospitalized for longer periods [3, 37]. In our study, the early use group stayed in the hospital for 26 days, whereas the late use group styed for 53.9 days. Early corticosteroid use within 10 days after diagnosis did not reduce mortality but reduced hospital stay compared to late corticosteroid use. Therefore, it is important to use corticosteroids in patients with severe COVID-19 at the most appropriate time.

Our study had several limitations. First, the sample size was small. It is important to note that the number of patients with severe COVID-19 in South Korea is relatively low compared to other countries around the world. For this reason, the sample size was limited, despite the participation of many tertiary hospitals in this study. To overcome this, we further analyzed clinical outcome and confirmed that it was meaningful (power of time from diagnosis to discharge, time from symptom to discharge, and lenth of hospital stay = 0.97, 0.967, and 0.997). The linear mixed model is exploratory, and further research is needed by increasing the sample size. Second, there was insufficient data on the clinical symptoms and missing laboratory findings. Third, since our study was performed retrospectively, some confounders could not be excluded. Both the dose and duration of corticosteroid use in the study varied for each patient. Fourth, the 10-day-based group was a relatively random request, and it should be further divided into subgroups in follow-up studies. Therefore, further large-scale studies in patients with severe COVID-19 are required. Fifth, the initial Ct values between the two groups were different. The difference in initial Ct values suggest that patients in the two groups may have different inflammation status, and this could have affected the outcomes in terms of steroids inhibiting inflammation. However, the lowest Ct value in the late use group was the same as that in the early use group. Sixth, most of the corticosteroids used in this study were methylprednisolone, rather than dexamethasone or hydrocortisone. Because the rationale for using dexamethasone is clear, many studies using other steroids have not been done. However recent studies have shown that methylprednisolone is not inferior to using dexamethasone [20].

Despite these limitations, our study provides valuable information on the clinical outcomes of using corticosteroids in patients with severe COVID-19. The results of this study supported the notion that corticosteroids can be a potential treatment option for severe COVID-19, and that it can be beneficial to use corticosteroids early within 10 days in severe COVID-19 patients.

## Conclusions

In conclusion, corticosteroids can be one of the treatment options for patients with severe COVID-19, and early use of corticosteroids showed favorable clinical outcomes which are related to a reduction in the length of hospital stay.

## Data Availability

All data and material collected in the current study are available from the corresponding author upon reasonable request.

## Abbreviations

COVID-19: Coronavirus disease 2019
SARS-CoV-2: Severe acute respiratory syndrome coronavirus 2
CRP: C-reactive protein
ESR: Erythrocyte sedimentation rate
Ct: Cycle threshold
SD: Standard deviation
